# COVID-19 Vaccine Hesitancy: A Cross-Sectional Study of Visible Minority Canadian Communities

**DOI:** 10.3390/vaccines13030228

**Published:** 2025-02-24

**Authors:** Candy Ochieng, Pammla Petrucka, George Mutwiri, Michael Szafron

**Affiliations:** School of Public Health, University of Saskatchewan, Saskatoon, SK S7N 5B5, Canada; candy.ochieng@usask.ca (C.O.); pmp139@mail.usask.ca (P.P.); george.mutwiri@usask.ca (G.M.)

**Keywords:** COVID-19, vaccine hesitancy, vaccine acceptance/refusal, vaccination coverage, preventive measures, public health communication

## Abstract

The World Health Organization (WHO) defines vaccine hesitancy as reluctance or refusal to vaccinate despite availability. Contributing factors in visible minority populations include vaccine safety, effectiveness, mistrust, socioeconomic characteristics, vaccine development, information circulation, knowledge, perceived risk of COVID-19, and perceived benefit. **Objectives**: This study aimed to examine vaccine hesitancy in visible minority populations across Canadian regions. **Methods**: A survey was conducted among visible minority populations in Canadian regions, using 21 questions from the available literature via the Delphi method. The Canadian Hub for Applied and Social Research (CHASR) administered the survey to individuals 18 years or older who resided in Canada at the time of the survey and identified as visible minorities such as Asian, Black, and Latin American. After recruiting 511 participants, data analysis used Chi-square tests of association and 95% confidence intervals (CIs) to identify regional differences in vaccine choices, side effects, information sources, and reasons for vaccination. A weighted analysis extended the results to represent the visible minorities across provinces. **Results**: Higher rates of Pfizer were administered to participants in Ontario (73%), the Prairies (72%), British Columbia (71%), and Quebec (70%). British Columbia had the highest Moderna rate (59%). The most common side effect was pain at the injection site in Quebec (62%), Ontario (62%), BC (62%), and in the Atlantic (61%). Healthcare professionals and government sources were the most trusted information sources, with healthcare professionals trusted particularly in the Prairies (70%) and government sources similarly trusted in Quebec (65%) and Ontario (65%). In the Atlantic, 86% of refusals were due to side effects and 69% were due to prior negative vaccine experiences. **Conclusions**: Leveraging healthcare professionals’ trust, community engagement, and flexible policies can help policymakers improve pandemic preparedness and boost vaccine acceptance.

## 1. Introduction

Vaccine hesitancy, as defined by the World Health Organization (WHO), is the reluctance or refusal to receive vaccines despite their availability, posing a significant threat to the progress against vaccine-preventable diseases [1]. To highlight the importance of vaccination, *The Lancet* in 2024 reported that worldwide immunization efforts have saved 154 million lives, or the equivalent of 6 lives every minute of every year for the last 50 years [2]. Medical providers identify vaccine hesitancy when patients request altered vaccination schedules, delay vaccines, or refuse them entirely, behaviours that often indicate underlying concerns about vaccinations [3,4]. Recognizing the challenges vaccine hesitancy poses to public health, the Strategic Advisory Group of Experts (SAGE) on Immunization established a dedicated Working Group in March 2012 to address this issue [5]. Factors influencing vaccine hesitancy have evolved over time, contributing to rising concerns regarding the COVID-19 vaccine [6]. A previous scoping review highlighted recurring contributors to vaccine hesitancy, including doubts about vaccine safety and effectiveness, mistrust, and socioeconomic characteristics [7]. Education and socioeconomic position are not consistently accurate predictors of vaccine hesitancy since their influence might fluctuate based on cultural, geographical, or contextual factors [8]. Amidst the COVID-19 pandemic, the introduction of new vaccines has intensified these issues, leaving many individuals still hesitant to receive the vaccine [9].

The WHO identifies complacency, access difficulties, and lack of confidence as major contributors to vaccine hesitancy [1]. The “3Cs” model classifies these factors into three broad categories, complacency, convenience, and confidence, while recognizing that individual experiences and cultural values shape how these factors affect different populations [8]. Despite reassurances from healthcare providers, many parents remain concerned about vaccine safety and thus hesitant to vaccinate their children [10]. Poor or inadequate communication, as well as factors like complacency and convenience, can also negatively impact vaccination uptake and increase hesitancy [8].

While common reasons such as mistrust in healthcare systems, misinformation, and perceived vaccine safety concerns often cause vaccine hesitancy, unique factors specific to COVID-19, such as the unprecedented speed of vaccine development, the politicization of vaccination campaigns, and rapidly evolving public health guidelines, also played a significant role [11]. There are examples in the literature that indicate visible minority populations have different responses towards vaccination. A study found that Black and Hispanic individuals in the United States are less likely than White individuals to trust the benefits of the measles, mumps, and rubella (MMR) vaccine, perceiving a higher risk of adverse effects [12]. Similarly, a cross-sectional study indicated that non-vaccination against COVID-19 was more common among off-reserve First Nations people (adjusted odds ratio [OR] 1.8, 95% confidence interval [CI] 1.2–2.7) and Black individuals (adjusted OR 1.7, 95% CI 1.1–2.6) but less common among South Asian individuals (adjusted OR 0.3, 95% CI 0.1–0.7) compared to White individuals [13]. Historical medical experimentation, racially segregated healthcare facilities, and ongoing prejudice have all contributed to eroding trust in medical institutions, and by extension, vaccination efforts [14,15]. These unique social and structural challenges and socioeconomic inequities likely influence the vaccine hesitancy observed in these groups [16]. While identifying the specific reasons for COVID-19 vaccine hesitancy among visible minority populations in Canada is crucial for reducing vaccine hesitancy and helping these populations, there is a dearth of studies in the literature regarding the Canada-wide impact on the behaviours and beliefs regarding COVID-19 vaccination among these groups [17]. This study aims to understand the regional variations among visible minority populations in Canada regarding their behaviours with respect to receiving a COVID-19 vaccination and its corresponding side effects as well as COVID-19 vaccination beliefs and information-seeking behaviours.

## 2. Methods

### 2.1. Survey Instrument

Based on the scoping review identifying known factors influencing COVID-19 vaccination in visible minorities across the globe [7], as part of this study, a survey tool was drafted to explore the aim of the study, and the file is available as a Supplementary Material. A Delphi approach was used to improve the survey instrument through integrating iterative feedback from a panel of experts [18]. The panel comprised five subject-matter experts and three graduate students. The finalized survey included questions regarding demographic information, types of COVID-19 vaccines received, side effects experienced post-vaccination, primary sources of COVID-19 information, reasons for vaccine refusal (if applicable), preventive measures to control the spread of COVID-19, and factors influencing vaccination decisions. The survey was conducted online in English between 9 May 2023 and 1 June 2023 and was facilitated by the Canadian Hub for Applied and Social Research (CHASR) at the University of Saskatchewan [19]. Ethical approval for the study was granted on 3 May 2023 by the Behavioural Research Ethics Board at the University of Saskatchewan (approval number: BEH-3972). CHASR administered the survey to individuals 18 years or older who resided in Canada at the time of the survey and identified as a visible minority (South Asian, Chinese, Black, Filipino, Latin American, Arab, Southeast Asian, West Asian, Korean, and Japanese). In total, 624 participants were sampled, of whom 511 completed the survey, yielding a completion rate of approximately 81.9%.

### 2.2. Statistical Analysis

The data analyses were conducted using IBM Corp. (2019). IBM SPSS Statistics for Windows, Version 26.0, Armonk, NY, USA and Microsoft Excel for Mac Version 16.85. The process started with data cleaning and recategorizing variables according to Statistics Canada regional classifications [20]. Gender, initially categorized as transgender, gender-neutral, non-binary, two-spirit, agender, pangender, genderqueer, those preferring to self-describe, and those preferring not to answer, was recategorized into male, female, and non-binary [20]. Provinces, except for Quebec, Ontario, and British Columbia, were recategorized into regions [20]: Atlantic (Newfoundland and Labrador, Prince Edward Island, Nova Scotia, and New Brunswick), Prairie (Manitoba, Saskatchewan, and Alberta), and Territories (Nunavut, Northwest Territories, and Yukon). Education status was recategorized as up to high school (less than high school or high school), some post-secondary (college diploma or technical or trade school), postgraduate (bachelor’s degree, master’s degree, or doctorate), and post-secondary education (program incomplete) [20]. Marital status was recategorized as married, common law (unchanged), widow/separated/divorced (grouped), and single/never married/in a relationship (grouped) [20]. Categorical responses were summarized in frequency tables (frequencies and percentages) and visualized via bar charts. Chi-squared tests of association and 95% confidence intervals (CIs) were used to identify regional differences in vaccine choice, side effects, information sources, preventive measures undertaken during the COVID-19 pandemic, and reasons for receiving a COVID-19 vaccination. The analyses explored regional differences in types of vaccines received, reactions post-vaccination, primary information sources, reasons for vaccine refusal, preventive measures taken, and factors influencing decisions for initial vaccinations and boosters. The analysis utilized a weighted sample extrapolated to represent the visible minority population across the regions described above.

## 3. Results

### 3.1. Demographics

**Figure 1:**
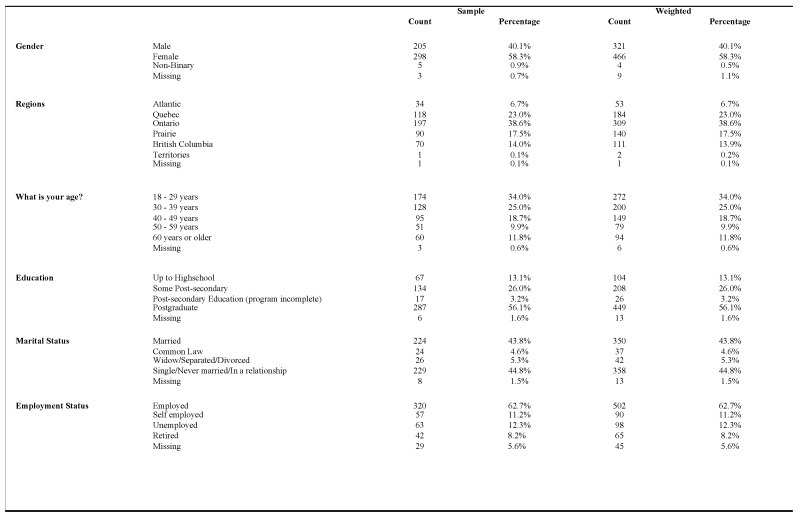
Recategorized frequency table of demographic variables.

The demographic information of the 511 study participants is summarized in Figure 1. The gender distribution of those who responded to the survey was 58.3% female participants, 40.1% male participants, and 0.5% non-binary participants. Ontario represented 38.6% of participants, and the Territories accounted for (0.2%). Vaccination rates by age group were as follows: 18–29 years (34%), 30–39 years (25%), 40–49 years (18.7%), 50–59 years (9.9%), and 60 years and above (11.8%). The number of participants holding postgraduate degrees (56.1%) followed closely with those with post-secondary education (26%). Marital status was nearly evenly split, with 43.8% of participants being married and 44.8% being single, never married, or in a relationship. Employment data revealed that approximately 62.7% of the sample were employed.

#### 3.1.1. Regional Variations in Types of COVID-19 Vaccines Received

**Figure 2:**
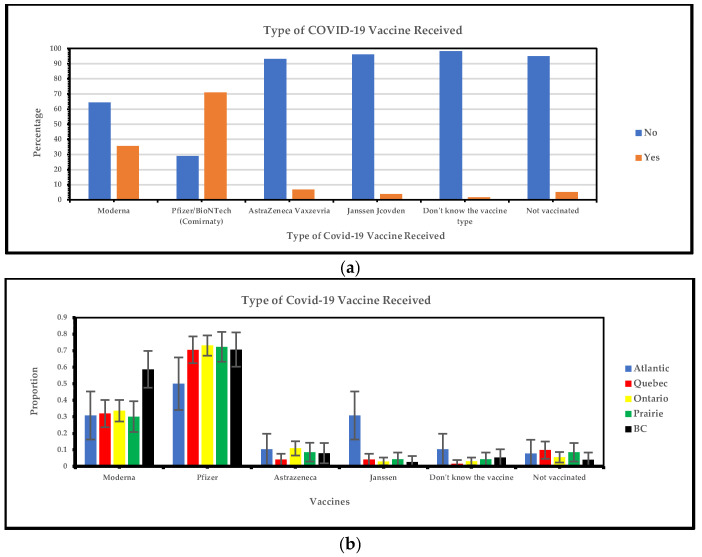
(**a**) Yes/no responses of type of COVID-19 vaccine received; (**b**) type of COVID-19 vaccine received.

Figure 2a below shows that the most frequent type of vaccine used is Pfizer and Moderna, the least frequent vaccines received are AstraZeneca and Janssen, and a small number of individuals comprised those who do not know the vaccine type and those who are not vaccinated. Figure 2b illustrates COVID-19 vaccine acceptability among vaccine-hesitant minority groups. The Pfizer vaccine had extensive coverage in Canada: Quebec (70%), Ontario (73%), the Prairies (72%), and British Columbia (71%). In contrast, coverage in the Atlantic provinces was notably lower (50%). Moderna administration was most prevalent in British Columbia, where 58.7% of participants reported receiving this vaccine.

#### 3.1.2. Regional Variations in Side Effects Experienced After Receiving COVID-19 Vaccines

**Figure 3:**
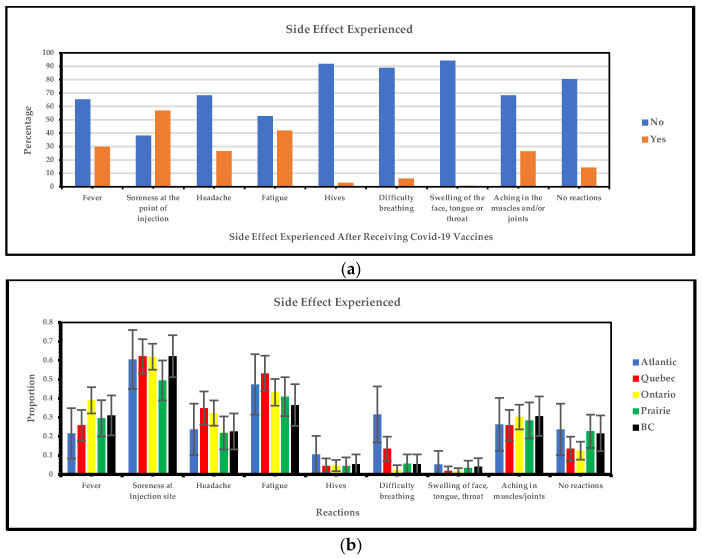
(**a**) Yes/no responses of side effects experienced after receiving COVID-19 vaccines; (**b**) side effects experienced after receiving COVID-19 vaccines.

Figure 3a below shows that the most frequent side effects are soreness at the injection site, fatigue, and fever, and the least frequent side effects are swelling of the face, tongue, and throat, hives, and difficulty breathing. Figure 3b illustrates region-specific adverse effects of COVID-19 vaccinations among vaccine-hesitant visible minority groups. Soreness at the injection site was the most common reaction reported by participants in the Atlantic provinces (61%), Quebec (62%), Ontario (62%), and British Columbia (62%), with a lower incidence in the Prairies (49%). Fever was most prevalent in Ontario, occurring in 39% of cases, while headaches in Quebec affected approximately 35% of participants, and in Ontario, about 32% of participants were affected.

#### 3.1.3. Regional Variations in Major Sources for Obtaining Information About COVID-19 Vaccines

**Figure 4:**
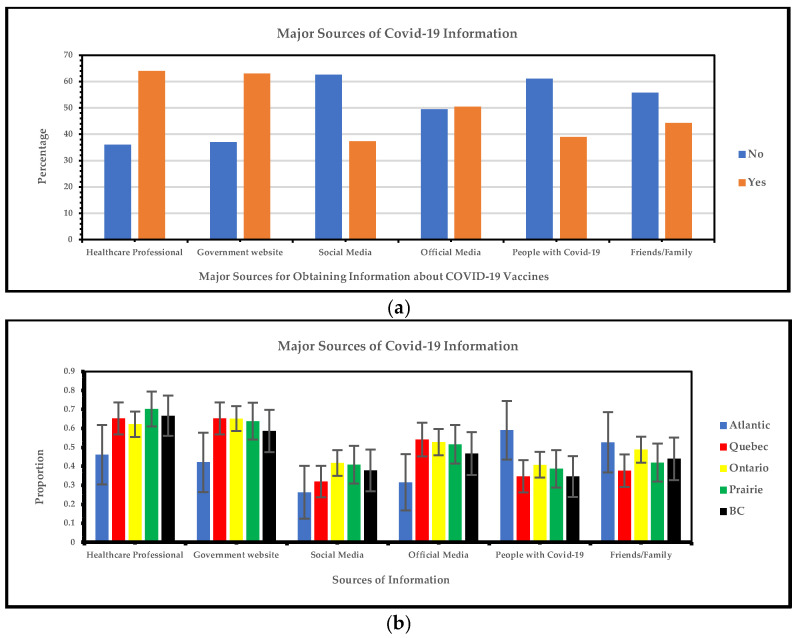
(**a**) Yes/no responses of major sources for obtaining information about COVID-19 vaccines; (**b**) major sources for obtaining information about COVID-19 vaccines.

Figure 4a below shows that the most frequent significant sources of COVID-19 information are healthcare professionals and government websites, and the least frequent sources are social media, people with COVID-19, and friends/family. Figure 4b highlights the primary sources of COVID-19 information, with healthcare professionals and government websites emerging as key resources across regions. In the Prairie region, 70% of individuals rely on healthcare professionals as their primary information source, followed closely by British Columbia (67%) and Quebec (65%). Government websites also show strong usage with minimal regional differences, with Quebec (65%), Ontario (65%), and the Prairie region (64%). By contrast, the Atlantic region shows a lower reliance on these sources, including healthcare professionals (46%), government websites (42%), social media (26%), and official media (32%).

#### 3.1.4. Regional Variations in Reasons for Refusing Recommended Vaccine by Healthcare Professionals: Relationship Analysis

**Figure 5:**
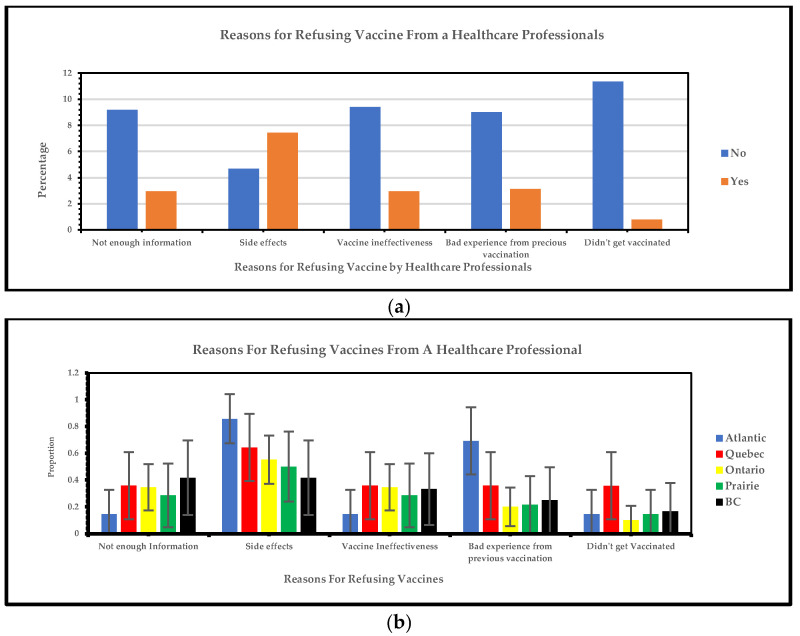
(**a**) Yes/no responses of reasons for refusing vaccine by healthcare professionals; (**b**) reasons for refusing vaccine by healthcare professionals.

Figure 5a below shows that the most frequent reasons for refusing a vaccine from a healthcare professional are side effects and bad experiences from previous vaccinations, and the least frequent reasons are participants who are not vaccinated, vaccine ineffectiveness, and insufficient information. Figure 5b shows that the Atlantic region reported a high prevalence of vaccine side effects (86%) and adverse events from previous immunizations (69%). Quebec had the second-highest response rates related to vaccine refusal, side effects (64%), and prior adverse events (36%). Additionally, the Atlantic region reported the lowest response rate for vaccine ineffectiveness (14%) as a reason for refusing the COVID-19 vaccine.

#### 3.1.5. Regional Variations in Measures Taken to Prevent COVID-19

**Figure 6:**
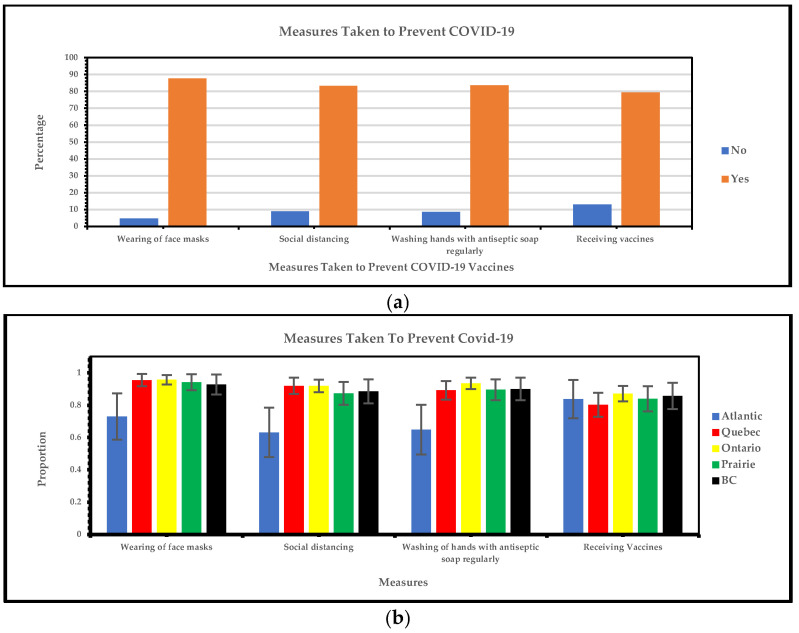
(**a**) Yes/no responses of measures taken to prevent COVID-19 vaccines; (**b**) measures taken to prevent COVID-19 vaccines.

Figure 6a below shows that the most frequent measures taken to prevent COVID-19 are wearing a face mask and washing hands with antiseptic soap regularly. The least frequent measures taken are receiving vaccines and social distancing. Figure 6b shows the adoption of COVID-19 preventative strategies such as wearing face masks, routinely washing hands with antiseptic soap, maintaining social distancing, and getting vaccinated across all regions. Response rates in Quebec, Ontario, the Prairie region, and British Columbia ranged from 80 to 95% with considerable overlap. However, the Atlantic region consistently showed lower rates for most preventative measures.

#### 3.1.6. Regional Variances in Sources Influencing Decision-Making for Initial COVID-19 Vaccination

**Figure 7:**
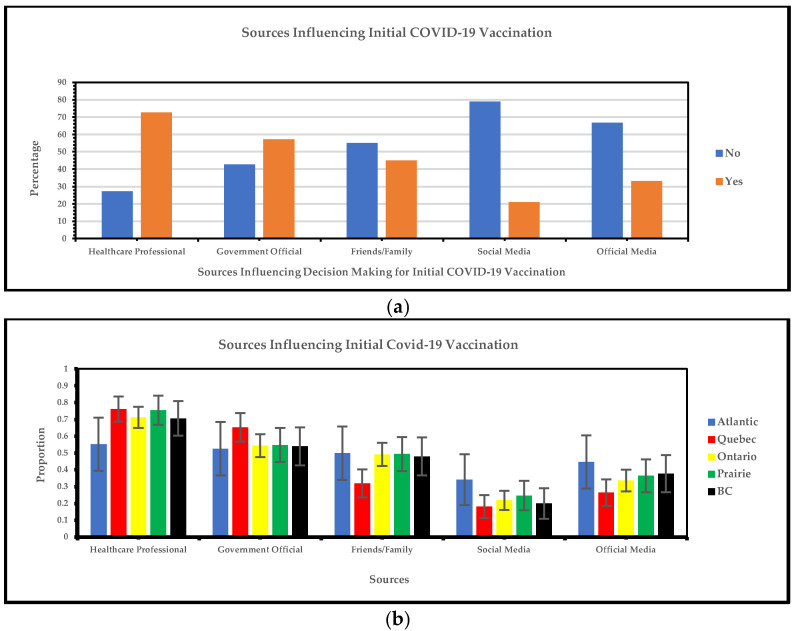
(**a**) Yes/no responses of sources influencing decision-making for initial COVID-19 vaccination; (**b**) sources influencing decision-making for initial COVID-19 vaccination.

Figure 7a shows that the most frequent sources influencing decision-making for initial COVID-19 vaccination are healthcare professionals and government websites, while the least frequent are social media, official media, and friends/family. Figure 7b illustrates the overlapping factors influencing initial COVID-19 vaccination decisions in the Prairie and British Columbia regions. Our study indicates that healthcare professionals are the primary influence on vaccination choices across all information sources, with Quebec leading at 76% and closely followed by the Prairie region (75%). Official government sources were the second most preferred source of information, again with Quebec in the lead (65%). Friends and family also had a notable but lesser impact compared to healthcare professionals and government officials, showing overlapping influence across the Atlantic (50%), Ontario (49%), the Prairies (49%), and the British Columbia region (48%). Social media had the least impact, with the Atlantic region leading at 34%.

#### 3.1.7. Regional Variations in Sources Influencing Decision-Making for Initial COVID-19 Booster: Relationship Analysis

**Figure 8:**
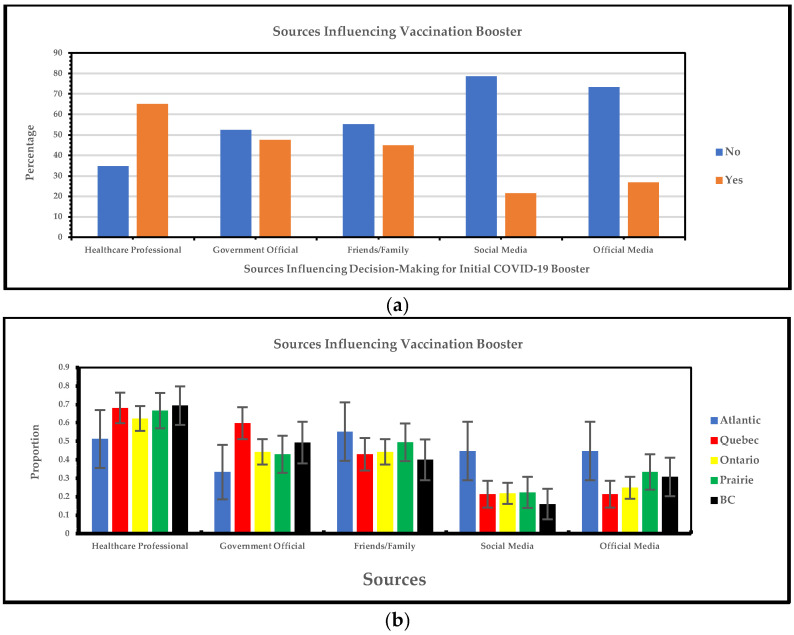
(**a**) Yes/no responses of sources influencing decision-making for initial COVID-19 booster; (**b**) sources influencing decision-making for initial COVID-19 booster.

Figure 8a shows that healthcare professionals, government websites, and friends and family are the most frequent sources influencing decision-making for initial COVID-19 vaccination boosters, while social media, official media, and friends and family are the least frequent. Figure 8b shows that vaccine-hesitant individuals in visible minority groups primarily rely on healthcare experts for guidance on vaccination boosters across all regions, with higher reliance in British Columbia (69%) and Quebec (68%). In contrast, the Atlantic region reports a higher reliance on friends and family (55%), social media (47%), and official media sources (45%), with both social media and official media sources response rates being 44%.

## 4. Discussion

This survey was conducted following a previous scoping review we published on factors associated with vaccine hesitancy in visible minority groups [7]. The global perspective of the review identified the safety and effectiveness of the vaccine, mistrust, and socioeconomic characteristics as the top three recurring factors using principal component analysis (PCA) [7]. Our current data collection aimed to examine how these themes relate to the responses of 511 survey participants from visible minority populations in Canada. This analysis not only reaffirmed the significance of these factors but also highlighted the importance of addressing specific regional and demographic variations to oppose vaccine hesitancy and improve public health outcomes.

Our survey data reveal that Pfizer is the most frequently received vaccine among the visible minority groups, followed by Moderna. The least frequently received vaccines are AstraZeneca and Janssen. As is known, the general population in Canada received Pfizer vaccines the most [21]. Our survey showed significant regional preferences for Pfizer/BioNTech (Comirnaty) and Moderna (Spikevax) vaccines in Canada. Both Pfizer-BioNTech and Moderna require at least two doses for full efficacy as well as AstraZeneca [1,22]. Moderna was administered to 35.5% of participants, primarily in Ontario and British Columbia (BC). In comparison, Pfizer/BioNTech was received by 71.1% of participants, showing widespread acceptance in Quebec, Ontario, the Prairies, and British Columbia. In contrast, AstraZeneca (Vaxzevria) and Janssen (Jcovden) had lower uptake rates of 6.9% and 3.8%, respectively; this can be supported by evidence of Pfizer on 9 October 2020, having been authorized as the first COVID-19 vaccine in Canada leading to its higher intake as compared to the rest of the vaccines [23]. Pfizer was frequently administered across Canada, with high uptake rates in Quebec, Ontario, the Prairies, and British Columbia but lower rates in the Atlantic region [22]. A supporting study revealed low variant of concern (VOC) prevalence and low vaccination rates in Nova Scotia (90 VOC cases per 1 million population, with a 5.6% vaccination rate), Prince Edward Island (175 VOC cases per 1 million population, with a 10.9% vaccination rate), New Brunswick (237 VOC cases per 1 million population, with a 10.5% vaccination rate), and Newfoundland and Labrador (374 VOC cases per 1 million population, with a 5.7% vaccination rate) [24]. Additionally, there was a preference for Moderna over Janssen, likely due to identified cases of thrombocytopenia syndrome (TTS) following Janssen vaccinations, including fatalities [25,26]. The higher vaccine uptake in British Columbia was attributed to the administration of three doses, indicating a consistent approach across regions in Canada [27]. Our survey advocates for better public health strategies by addressing regional preferences and concerns and understanding the widespread acceptance of Moderna and Pfizer/BioNTech, particularly in Quebec, Ontario, the Prairies, and British Columbia, which can enhance vaccine distribution efforts among visible minority groups in Canada. Addressing regional differences and maximizing trust in accepted vaccines will make public health campaigns more effective in increasing vaccination rates and managing future health crises.

Our survey shows that the most frequent side effects are soreness at the injection site, fatigue, and fever, and the least frequent side effects are swelling of the face, tongue, and throat, hives, and difficulty breathing. The analysis of side effects among visible minority populations following COVID-19 vaccinations revealed that soreness at the injection site is the most consistently reported reaction across all regions, indicating that it is a typical and reliably measured response. Soreness is a typical, expected reaction regardless of location. These findings align with national adverse event surveillance data. According to Health Info Base, as of 19 January 2024, soreness at the injection site and mild fever were among the most frequently reported side effects [21]. Additionally, Adverse Event Following Immunization (AEFI) reports from Public Health Ontario indicated that, out of 4147 total adverse events reported across all vaccines, soreness at the injection site was the most frequently cited, surpassing other reactions like rash and severe vomiting [28]. Since government monitoring data match participant-reported adverse effects, participant comments may be verified. Minor adverse effects, including discomfort and heat, are common, but significant side effects are rare. Evidence-based healthcare initiatives may educate individuals about common and expected adverse effects to promote public health and encourage vaccination. Specific group education initiatives can dispel vaccine myths and boost trust. Also, making follow-up care and medical advice easy to receive may help vaccine-hesitant people accept them. Standardized vaccine distribution and a way to monitor and report issues can make immunization programs more transparent. By addressing these factors, public health initiatives can more effectively tackle vaccine hesitancy and build trust among visible minority populations.

Our study identified the most frequent sources of COVID-19 information in visible minorities to be healthcare professionals and government websites, and the least frequent sources to be social media, people with COVID-19, and friends/family. A 2020 Canadian cross-sectional study identified that Canadian television, magazines, and non-governmental websites were the most trusted sources of COVID-19 information for the general Canadian population [29]. Since the onset of the pandemic, COVID-19 information has circulated widely online and on social media, leading many Canadians to rely on these platforms despite the risk of misinformation [30]. Our survey reveals that visible minority individuals prefer receiving COVID-19 information from healthcare professionals and government websites. Campaigns targeting these sources may help counter misinformation and improve future vaccine uptake.

As our study shows, the most frequent reasons visible minorities refuse a vaccine from a healthcare professional are side effects and bad experiences from previous vaccinations, and the least frequent reasons are participants who are not vaccinated, vaccine ineffectiveness, and insufficient information. A Canadian study showed that about 89% reported at least one side effect on the initial COVID-19 vaccines, and 11% of participants across groups reported no side effects. The reported effects were tiredness/fatigue, muscle/body pain, headache, chills, fever, nausea, and abdominal pain [31]. Another study showed that pain at the injection site, fatigue, and headaches were some of the significant side effects of receiving COVID-19 vaccines [29]. According to our study, the Atlantic region reported a high rate of side effects and adverse experiences from previous vaccines despite having the lowest instances of other reasons for refusing the COVID-19 vaccine. The Atlantic region continues to exhibit significant policy variation, with measures adapted to suit more localized contexts through zones and tiered classification systems [32]. This initiative helped mitigate the spread of COVID-19 [32] and dramatically influenced this region’s low turnout for refusing the vaccines [32]. The decline in most cases in the Atlantic region may be attributed to the “Atlantic bubble” within the four provinces (Nova Scotia, New Brunswick, Prince Edward Island, and Newfoundland and Labrador) [32]. The successful implementation of localized measures in the Atlantic region can serve as a model for other regions in Canada. Policymakers could adopt similar strategies, such as creating localized zones and tiered classification systems, to manage public health responses during future pandemics. Additionally, forming regional bubbles, as seen with the “Atlantic bubble,” could contain the spread of infectious diseases while minimizing disruption to social and economic activities. These approaches could help other regions enhance their pandemic preparedness and response efforts among visible minority groups [32].

In the general Canadian population, on 11 March 2020, social distancing was one of the preventive measures implemented to control the spread of COVID-19 [33,34,35]. As the pandemic continued, social distancing was still imposed across Canada. We found that among visible minorities, a combination of strategies was used to mitigate the spread of COVID-19. We identified that the most frequent measures taken to prevent COVID-19 were wearing a face mask, social distancing, and washing hands with antiseptic soap regularly, and the least frequent measure was being vaccinated. We found that Quebec, Ontario, and the Prairie regions reported similar proportions for these measures, while the Atlantic region showed lower rates for the uptake of most preventive actions. Similarly, in the general Canadian population, a variety of measures were used to prevent the spread of COVID-19: restrictive measures, social distancing, lockdowns, case detection, isolation, contact tracing, and quarantining exposed individuals [36]. Similar to our study, the general Canadian population in the Atlantic region had a lower response rate to these measures [32]. As a result, there were fewer cases in these regions, leading some individuals to be less rigorous with preventive actions compared to other parts of Canada [32]. Policymakers should create localized approaches to pandemic responses, adopt a combination of strategies, and ensure that policies are adaptable to local contexts. The “Atlantic bubble” can help other regions enhance their preparedness and response efforts, ultimately leading to more effective control of disease spread across the country.

Our survey showed that healthcare professionals, government websites, and friends and family were the most frequent sources influencing decision-making for initial COVID-19 vaccination and boosters and social media, official media, and friends and family were the least frequent. Initial COVID-19 vaccines were influenced by sources like television, pamphlets, and healthcare professionals [37]. The most frequent sources that influenced the general Canadian population to take COVID-19 boosters were government decision-making processes and healthcare providers [38], followed by personal or family reasons [39]. A cross-sectional study from Canada showed that some factors, such as disagreeing with government decision-making (somewhat disagree: OR 2.70, CI 1.38, 5.29; strongly disagree: OR 4.62, CI 2.20, 9.7), were associated with booster dose hesitancy [40]. The comparison between visible minorities and the general population regarding primary sources influencing initial and COVID-19 uptake along with the booster differs, with the general population in Canada frequently using television to influence them in getting the COVID-19 vaccine [35]. At the same time, our study showed that official media sources were the least used among the visible minority group. Our work identifies sources that can be targeted to describe the importance of vaccination to improve vaccine uptake among visible minority groups in Canada.

## 5. Conclusions

This study aims to understand the regional variations among visible minority Canadian populations regarding their behaviours in receiving COVID-19 vaccination, its corresponding side effects, and their COVID-19 vaccination beliefs and information-seeking behaviours. Our survey revealed that in visible minority populations, significant sources of COVID-19 information were healthcare and government websites while Canadian television and governmental websites were significant in the general Canadian population. Preventive measures frequently used by visible minorities were face masks and hand washing regularly with antiseptic soap, followed by social distancing, while the general Canadian population prioritized social distancing. As for the initial COVID-19 vaccination, our study revealed that the most frequent sources were healthcare professionals for minorities, and for the general Canadian population, television (unofficial media), initial COVID-19 vaccination, and the government website were sources of information on COVID-19 boosters. We show how information sources, trust levels, and views about the safety of vaccinations vary by region and how important it is to use different communication methods. Public health officials should tailor vaccine messaging to each group, and to solve these problems, we need clear communication that focuses on specific community issues, early participation of underrepresented groups, and public health policies that can change to account for differences in population. These changes will improve the likelihood of public health officials getting people to accept vaccines more readily in the future.

## Data Availability

The datasets are not readily available due to privacy concerns. Requests to access de-identified datasets should be directed to the corresponding author.

## Supplementary Materials

The following supporting information can be downloaded at: https://www.mdpi.com/article/10.3390/vaccines13030228/s1. The supplementary material for this study includes the questionnaire used for data collection. It provides details on the survey items and structure. References [41,42,43,44,45,46,47,48,49,50,51,52] are cited in the Supplementary Materials.
