# Supplementary material for: COVID-19 Vaccine Hesitancy: A Cross-Sectional Study of Visible Minority Canadian Communities
Source: Vaccines (Basel). 2025 Feb 24;13(3):228. doi: 10.3390/vaccines13030228 (PMC11946865; doi:10.3390/vaccines13030228)
Supplement: Supplementary file 1 [file vaccines-13-00228-s001.zip › vaccines-3387705-supplementary.pdf]

CONSENT

UNIVERSITY OF SASKATCHEWAN

Online Participant Consent Form

[Insert Ethics Approved Consent Form]

SCREEN1
What is your ethnicity?

- ☐ Asian
- ☐ Black/African/Caribbean
- ☐ Hispanic/Latino
- ☐ Middle Eastern
- ☐ Indigenous
- ☐ Métis
- ☐ Inuit
- ☐ White/Caucasian/European

SCREEN2
Do you consider yourself an ethnic minority?

The following groups make up most of the visible minority population: South Asians, Chinese, Blacks, Filipinos, Latin Americans, Arabs, Southeast Asians, West Asians, Koreans, and Japanese.

- ○ Yes
- ○ [illegible]

○ No

Q3
With what gender do you identify?

○ Man

○ Woman

○ Transgender

○ Gender neutral

○ Non-binary

○ Two-spirit

○ Agender

○ Pangender

○ Gender queer

○ Prefer to self-describe as (please specify): [ ]

○ Prefer not to answer

Q4
What is your age?

○ 18 - 29 years

○ 30 - 39 years

○ 40 - 49 years

○ 50 - 59 years

○ 60 years or older

○ Prefer not to answer

Q5
What is the highest level of education you have completed?

- ○ Less than high school
- ○ High school
- ○ Diploma
- ○ College, Technical, or Trade School
- ○ Bachelors degree
- ○ Master's degree
- ○ Doctorate degree
- ○ Post-secondary education (program incomplete)
- ○ Other (please specify) ____________
- ○ Prefer not to answer

Q6
What is your marital status?

- ○ Single
- ○ In a relationship
- ○ Common-law
- ○ Married
- ○ Divorced
- ○ Widowed
- ○ Other (please specify) ____________
- ○ Prefer not to answer

Q7
What is your current employment status?

- ○ Employed
- ○ Self employed
- ○ Unemployed
- ○ Retired
- ○ Other (please specify) ________
- ○ Prefer not to answer

Q8
What type of COVID-19 vaccine(s) have you received? Check all that apply.

If you have not received a COVID-19 vaccine, please indicate so.

- ☐ Moderna (Spikevax)
- ☐ Pfizer/BioNTech (Comirnaty)
- ☐ AstraZeneca Vaxzevria
- ☐ Janssen Jcovden (Johnson & Johnson)
- ☐ Other (please specify) ________
- ☐ I have been vaccinated but do not know the vaccine type
- ☐ I have not been vaccinated
- ☐ Prefer not to answer

Q9
What, if any, reactions did you experience after receiving the COVID-19 vaccine(s)? Check all that apply.

- ☐ Fever
- ☐ Soreness at the point of injection
- ☐ Headache
- ☐ Fatigue
- ☐ Hives (bumps on the skin that are usually itchy)
- ☐ Difficulty breathing
- ☐ Swelling of the face, tongue or throat
- ☐ Aching in the muscles and/or joints
- ☐ Other (please specify) ______
- ☐ I did not experience any reactions
- ☐ Prefer not to answer

Q10
How would you rate your knowledge of COVID-19?

- ○ Very good
- ○ Good
- ○ Average
- ○ Poor
- ○ Very poor

Q11
Which of the following were your major sources for obtaining information about COVID-19? Check all that apply.

- ☐ Healthcare professionals
- ☐ Government website
- ☐ Social media (Facebook, Twitter, Instagram, Snapchat, etc.)
- ☐ Official media (News channel, radio stations, newspapers, etc.)
- ☐ People who have had COVID-19
- ☐ Friends/family
- ☐ Other (please specify) ____
- ☐ None of the above
- ☐ Prefer not to answer

Q12
To what extent do you trust the process of COVID-19 vaccine development?

The vaccine process means the manufacturing of the COVID-19 vaccine with factors that make it safe and effective for human administration)

- ○ Entirely
- ○ Mostly
- ○ Somewhat
- ○ Not really
- ○ Not at all

Q13
Have you ever refused a vaccine that was recommended to you by a healthcare professional?

- ○ Yes
- ○ No

Q14
What was the reason(s) for refusing a vaccine recommended by a healthcare professional? Check all that apply.

- ☐ I did not have enough information on the vaccine
- ☐ I was concerned about side effects
- ☐ I did not think the vaccine was effective
- ☐ I had a bad experience with a previous vaccination
- ☐ I did not know where to get vaccination
- ☐ Other (please specify) ______
- ☐ Prefer not to answer

Q15
Do you think that your family members should be vaccinated against COVID-19?

- ○ Definitely should be
- ○ Probably should be
- ○ Maybe
- ○ Probably should not be
- ○ Definitely should not be

Q16
Would you accept the COVID-19 vaccine if it were administered by an oral or non-injectable route?

Non-injectable route: this method involves injecting the vaccine intramuscularly into the muscle through the skin and subcutaneous tissue using a needle.

- ○ Yes
- ○ No
- ○ Maybe
- ○ Uncertain
- ○ Prefer not to answer

Q17
Has social media (Facebook, Twitter, Instgram, Snapchat, etc.) influenced your perceptions towards receiving the COVID-19 vaccination?

- ○ Yes (please specify the media source(s) that had influence) ______
- ○ No
- ○ Prefer not to answer

Q18
Has official media (news channels, radio stations, newspapers, etc.) influenced your perceptions towards receiving the COVID-19 vaccination?

- ○ Yes (please specify the media source(s) that had influence) ______
- ○ No
- ○ Prefer not to answer

Q19
Did you take any measures to prevent COVID-19?

- ○ Yes
- ○ No

Q20
Please indicate which measures you have taken to prevent COVID-19. Check all that apply.

- ☐ Wearing of face masks
- ☐ Social distancing
- ☐ Washing of hands regularly with an antiseptic soap
- ☐ Receiving vaccine(s)
- ☐ Other (please specify) ______
- ☐ Prefer not to answer

Q21
If a medical professional recommended the vaccine, how likely are you to receive an initial COVID-19 vaccination?

- ○ Definitely will
- ○ Probably will
- ○ Maybe
- ○ Probably won't
- ○ Definitely won't

Q22
If a medical professional recommended the vaccine, how likely are you to receive a COVID-19 booster?

- ○ Definitely will
- ○ Probably will
- ○ Maybe
- ○ Probably won't
- ○ Definitely won't

Q23
What sources influenced your decision to get or not to get an initial Covid-19 vaccination? Check all that apply.

- ☐ Healthcare professionals
- ☐ Government officials
- ☐ Friends/family
- ☐ Social media (Facebook, Twitter, Instagram, Snapchat, etc.)
- ☐ Official media (News channel, radio stations, newspapers, etc.)
- ☐ Other (please specify) ____________
- ☐ Prefer not to answer

Q24
What sources influenced your decision to get or not to get a Covid-19 booster? Check all that apply.

- ☐ Healthcare professionals
- ☐ Government officials
- ☐ Friends/family
- ☐ Social media (Facebook, Twitter, Instagram, Snapchat, etc.)
- ☐ Official media (News channel, radio stations, newspapers, etc.)
- ☐ Other (please specify) ______
- ☐ Prefer not to answer

Q25
Do you have a preferred COVID-19 vaccination type? (e.g., Pfizer, Johnson/Johnson, AstraZeneca, Moderna)

- ○ Yes (please specify which one) ______
- ○ No
- ○ Prefer not to answer

References

1. Fisher, K.A.; Bloomstone, S.J.; Walder, J.; Crawford, S.; Fouayzi, H.; Mazor, K.M. Attitudes toward a potential SARS-CoV-2 vaccine: A survey of US adults. Ann. Intern. Med. 2020, 173, 964–973.
2. Gatwood, J.; McKnight, M.; Fiscus, M.; Hohmeier, K.C.; Chisholm-Burns, M. Factors influencing likelihood of COVID-19 vaccination: A survey of Tennessee adults. Am. J. Health-Syst. Pharm. 2021, 78, 879–889.
3. Ogilvie, G.S.; Gordon, S.; Smith, L.W.; Albert, A.; Racey, C.S.; Booth, A.; Gottschlich, A.; Goldfarb, D.; Murray, M.C.; Galea, L.A.; et al. Intention to receive a COVID-19 vaccine: Results from a population-based survey in Canada. Bmc Public Health 2021, 21, 1017.
4. Reuben, R.C.; Danladi, M.M.; Saleh, D.A.; Ejembi, P.E. Knowledge, attitudes and practices towards COVID-19: An epidemiological survey in North-Central Nigeria. J. Community Health 2021, 46, 457–470.
5. Wong, M.C.; Wong, E.L.; Huang, J.; Cheung, A.W.; Law, K.; Chong, M.K.; Ng, R.W.; Lai, C.K.; Boon, S.S.; Lau, J.T.; et al. Acceptance of the COVID-19 vaccine based on the health belief model: A population-based survey in Hong Kong. Vaccine 2021, 39, 1148–1156.
6. Kweon, S.S.; Yun, I.; Choi, C.; Ryu, S.Y.; Cho, J.H.; Shin, M.H. Factors associated with COVID-19 vaccine hesitancy in Korea. Chonnam Med. J. 2022, 58, 43.
7. Leigh, J.P.; Moss, S.J.; White, T.M.; Picchio, C.A.; Rabin, K.H.; Ratzan, S.C.; Wyka, K.; El-Mohandes, A.; Lazarus, J.V. Factors affecting COVID-19 vaccine hesitancy among healthcare providers in 23 countries. Vaccine 2022, 40, 4081–4089.
8. Skeens, M.A.; Hill, K.; Olsavsky, A.; Buff, K.; Stevens, J.; Akard, T.F.; Shah, N.; Gerhardt, C.A. Factors affecting COVID-19 vaccine hesitancy in parents of children with cancer. Pediatr. Blood Cancer 2022, 69, e29707.
9. Tsai, R.; Hervey, J.; Hoffman, K.; Wood, J.; Johnson, J.; Deighton, D.; Clermont, D.; Loew, B.; Goldberg, S.L. COVID-19 vaccine hesitancy and acceptance among individuals with cancer, autoimmune diseases, or other serious comorbid conditions: Cross-sectional, internet-based survey. JMIR Public Health Surveill. 2022, 8, e29872.
10. Nazlı, Ş.B.; Yığman, F.; Sevindik, M.; Deniz Özturan, D. Psychological factors affecting COVID-19 vaccine hesitancy. IRISH J. Med. Sci. (1971-) 2022, 191, 71–80.
11. Santirocchi, A.; Spataro, P.; Costanzi, M.; Doricchi, F.; Rossi-Arnaud, C.; Cestari, V. Predictors of the Intention to Be Vaccinated against COVID-19 in a Sample of Italian Respondents at the Start of the Immunization Campaign. J. Pers. Med. 2022, 12, 111.
12. Alabdulla, M.; Reagu, S.M.; Al-Khal, A.; Elzain, M.; Jones, R.M. COVID-19 vaccine hesitancy and attitudes in Qatar: A national cross-sectional survey of a migrant-majority population. Influenza Other Respir. Viruses 2021, 15, 361–370.
